# Shedding light on local kinase activation

**DOI:** 10.1186/1741-7007-10-61

**Published:** 2012-07-17

**Authors:** John D Scott, Alexandra C Newton

**Affiliations:** 1 Howard Hughes Medical Institute, Department of Pharmacology, University of Washington School of Medicine,1959 Pacific Ave. NE, Seattle, WA 98195 USA; 2 Department of Pharmacology, University of California at San Diego, 9500 Gilman Drive, La Jolla, CA 92093, USA

## Abstract

Phosphorylation is the predominant language of cell signaling. And, as with any common language, an abundance of dialects has evolved to convey complex information. We discuss here how biosensors are being used to decode this language, affording an unprecedented glimpse into spatio-temporal patterns of protein phosphorylation events within the cell.

## Opinion

Intracellular protein kinases relay information that originates from diverse chemical and ionic signals received at the cell surface, phosphorylating their substrates to elicit a variety of responses. A relatively small group of second messenger molecules, and the kinases they activate, function in a plethora of different signaling pathways, posing the question of how the specificity of the original signal is not lost in transmission. One answer to this appears to be the routing of signals with spatial fidelity and tight temporal control, through signaling complexes brought together on protein scaffolds. As we illustrate in this article, direct insights into local patterns of kinase activation have been made possible by the ingenious development of genetically encoded kinase activity reporters. These biosensors translate the language of cell signaling, as it occurs in real time, into observable bursts of light.

## How light can report on a signaling event

**Figure 1:**
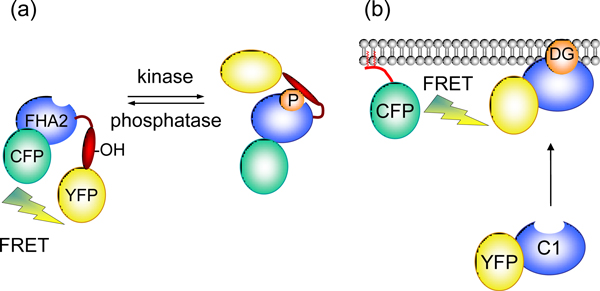
**The architecture of kinase activity reporters**. **(a) **Illustration of the modular architecture used for BKAR, CKAR, and DKAR. The reporter comprises cyan and yellow variants of GFP (CFP and YFP) flanking the phospho-threonine-binding FHA2 domain from the checkpoint kinase rad53 (blue) fused to a substrate sequence (red) for the relevant kinase by a flexible linker. Phosphorylation of the substrate segment causes an intramolecular rearrangement resulting from binding of the FHA2 domain to the phosphorylated substrate sequence (phosphate represented by orange circle). This conformational change alters the amount of FRET from CFP to YFP. The conformational change is readily reversed by dephosphorylation of the substrate segment by cellular phosphatases. The FRET change allows ratiometric measurement of CKAR phosphorylation in cells, revealing the dynamic balance between kinase and phosphatase activities in real time. Note that for other reporters (for example, AKAR, which uses a different phosphopeptide binding module) phosphorylation results in an increase in FRET. **(b) **A variation of this theme depicting intermolecular FRET used to measure the generation of diacylglycerol (DG) at any membrane of interest. The donor is CFP fused to a targeting sequence for the membrane of interest and the acceptor is the C1 domain, a diacylglycerol-sensing module, fused to YFP.

Nature has an abundance of fluorescent proteins that absorb and emit light [1]. Since these fluorescent proteins, or chromophores, have unique excitation/emission properties, each can be independently detected. Moreover, they can be paired in such a way that, when brought into close proximity, it is possible to detect energy transfer between them if the emission spectrum of one - the donor - overlaps with the absorption spectrum of the other - the acceptor. The elegant but simple logic behind 'biosensors' is to design a light-activated protein so that it responds to an altered biochemical parameter - whether it be phosphorylation, second messenger synthesis or recruitment of a binding partner - with detectable changes in the light spectrum emitted. Additionally, other elements of the biosensor are designed so that a change in the biological parameter of interest induces an intramolecular change, and thereby alters fluorescence resonance energy transfer (FRET) from the donor to the acceptor chromophore (Figure 1). A common donor-acceptor pair comprises monomeric variants of cyan fluorescence protein (for example, Cerulean (CFP); excitation/emission 433/475 nm) and yellow fluorescent protein (for example, mCitrine (YFP); excitation/emission 516/529 nm) that transfer energy when they are less than 10 nm apart. Thus, changes in the ratio of CFP to YFP emission from biosensors expressed in living cells report on a biological activity. FRET sensors can report on phosphorylation by specific kinases, and in this article we shall mainly focus on those designed to monitor the activity of the second messenger responsive serine/threonine kinases A, B, C and D as they function in a variety of biologically important signaling pathways. FRET sensors are also ideal to measure the dynamics of cAMP, Ca^2+ ^and lipid second messenger responses. Accordingly, this technology offers a relatively non-invasive way to monitor the action of diffusible chemical signals or protein phosphorylation events in real time.

The concept of using genetically encoded fluorescent reporters to visualize kinase signaling was pioneered by Tsien and others, who constructed kinase activity reporters for the epidermal growth factor receptor and for protein kinase A (called AKAR for A-kinase activity reporter) using the modular structure shown in Figure 1a[1-3]. These reporters serve as surrogate substrates of the kinase. They comprise a donor-acceptor pair (typically CFP and YFP) flanking a module that contains a phosphorylation sequence directed towards the relevant kinase, and a phosphopeptide-binding module that recognizes the phosphorylated sequence. Phosphorylation-dependent changes in FRET, as measured by changes in the ratio of YFP/CFP emission, report fluctuations in cellular kinase activity. This basic modular framework was subsequently used for the design of BKAR, CKAR and DKAR, a series of reporters that monitor the activities of protein kinases B, C and D, respectively [4-6]. Collectively, these 'KAR' reporters have afforded investigators unprecedented insight into the rate, amplitude, and duration of agonist-evoked kinase signaling. For example, CKAR revealed oscillations in PKC activity that are phase-locked with Ca^2+ ^oscillations [4].

Genetically encoded FRET reporters have also been developed to measure dynamic changes in second messenger levels [7,8]. These take advantage of nature's repertoire of modules that specifically bind the second messenger of interest. Figure 1b shows an example of how intermolecular FRET can be use to measure the generation of diacylglycerol at any membrane of interest. Here, the donor is CFP fused to a targeting sequence for the membrane of interest (for example, the plasma membrane) and the acceptor is the C1 domain, a diacylglycerol-sensing module, fused to YFP. This YFP-C1 module is recruited to all membranes containing diacylglycerol, but FRET will only occur on the membrane surface containing the CFP donor. Thus, location-specific generation of diayclglycerol can be measured. This reporter has revealed that diacylglycerol production at the Golgi is sustained, resulting in sustained activation of protein kinase C (PKC) at this membrane [9].

## Monitoring site-specific signaling activity

Because these signaling biosensors are genetically encoded, subcellular targeting motifs can be incorporated into their modular structure to direct these reporters toward organelles and intracellular membranes. Such targeted reporters have been useful to monitor the rates of signal transmission and kinase activity at the plasma membrane, Golgi, mitochondria, and recently in the nucleus [5,10,11]. For example, compartment-specific biosensors have also been used to estimate the diffusion rates of cAMP from its site of production at the plasma membrane into the nucleus [12]. A variation on this theme has been utilization of evanescent-wave fluorescence imaging (also called total internal reflected fluorescence or TIRF) to detect real-time changes in cAMP accumulation just beneath the plasma membrane: this method measures FRET between a truncated regulatory (RII) subunit of protein kinase A (PKA) fused to a farnesylated-CFP (to tether to the plasma membrane) and the PKA catalytic (C) subunit fused to YFP [13]. Holoenzyme dissociation in response to cAMP is monitored as PKA C-YFP translocates into the cytoplasm when released from the complex. These studies suggest that insulin release from glucose-stimulated β-cells is pulsatile and driven by oscillations in cAMP production [14,15].

**Figure 2:**
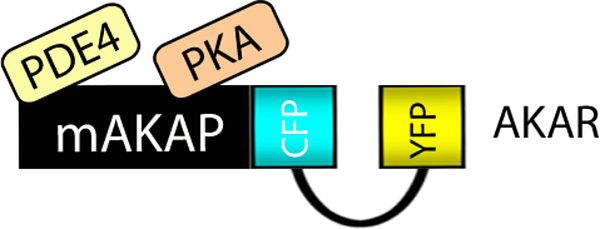
***In situ *detection of kinase activity with genetically encoded FRET reporter AKAR**. A schematic diagram of the AKAR kinase activity reporter fused to the PKA and PDE4 binding sites of the A Kinase anchoring protein, mAKAP. The fluorescent moieties (CFP and YFP) are indicated. This FRET sensor detects changes in intracellular cAMP.

Another means to monitor compartmentalized kinase activity is to fuse biosensors to scaffolding proteins. The best-characterized class of kinase scaffolds are the A-Kinase Anchoring proteins (AKAPs) that compartmentalize the cAMP-dependent protein kinase (PKA) to defined locations in the cell [16,17]. Thus, fusion of kinase activity reporters to AKAPs provides unique tools to sample local kinase activity. This is illustrated by an early study on signaling coordinated on the muscle-specific anchoring protein mAKAP. This AKAP spatially organizes PKA in proximity to type 4 phosphodiesterases (PDE4), an enzyme family that metabolizes cAMP to terminate the second messenger response. Such a configuration was mimicked in the context of an AKAR-PKA-PDE reporter where PKA and PDE4 binding sites derived from mAKAP were fused at either end of the FRET sensor [18] (Figure 2). This construct mimics mAKAP as it detects rapid and transient FRET responses that depict PKA activation upon hormonal elevation of intracellular cAMP. Importantly, these dynamic changes in PKA activity were prevented when the same cell was treated with the PDE4 inhibitor rolipram [18]. These control experiments confirm that recruitment of PDE4 to the mAKAP signaling complex terminates anchored PKA activity within that cellular microenvironment.

**Figure 3:**
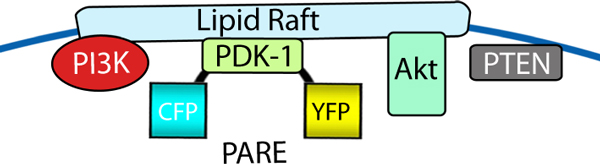
***In situ *detection of phosphatidylinositol-3-kinase pathway activity in lipid rafts with the FRET reporter PARE**. A schematic diagram of the PARE kinase activity reporter fused with the PDK-1 sequence. The FRET-based PDK-1 activation reporter, PARE, serves as a sensor for the phosphatidylinositol-3-kinase (PI3K), PDK-1 activation in lipid rafts. These upstream elements activate protein kinase B/Akt in a variety of cell types.

Protein kinase B (PKB) activity is also under acute spatiotemporal control and this has been visualized by BKAR [5]. Although protein scaffolds for PKB (also known as Akt) are not well defined, the role of compartmentalization in controlling how PKB propagates signaling in the phosphatidylinositol-3-kinase (PI3K) pathway is elegantly unveiled in the recent work of Zhang and coworkers [19]. Using reporters that have an improved sensitivity over the earlier BKAR and a new FRET-based phosphoinositide-dependent kinase-1 (PDK-1) activation reporter (PARE; Figure 3), these authors show that the machinery to propagate signaling in the PI3K pathway is coordinated in lipid rafts and that the machinery to terminate signaling in this pathway is excluded from lipid rafts. For this study, PDK-1 was engineered to have a FRET pair flanking the PDK-1 sequence, such that conformational changes attendant to activation could be monitored by FRET changes. Using this new reporter, growth factors were shown to activate PDK-1 in lipid rafts where its target, Akt/PKB, is also activated. Thus, both PDK-1 activity and Akt/PKB activity were localized on membrane rafts. In contrast, the lipid phosphatase PTEN, which negatively regulates the PI3K pathway by dephosphorylating the lipid second messenger phosphatidylinositol (3,4,5)-trisphosphate, is localized in non-raft regions. Zhang and coworkers [19] showed that mislocalizing PTEN to lipid rafts abolished growth factor signaling in the PI3K pathway. This work underscores the critical role played by compartmentalizing kinases and phosphatases for normal signaling, and how disruption of this compartmentalization leads to pathologies.

**Figure 4:**
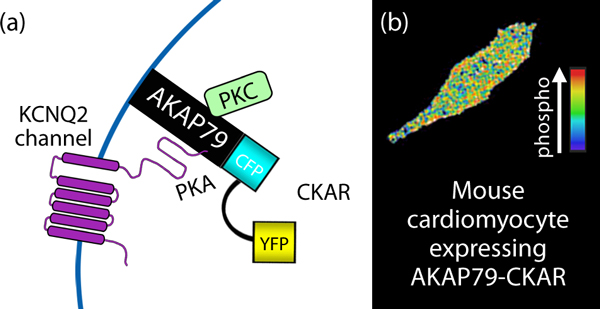
***In situ *detection of kinase activity with genetically encoded FRET reporter CKAR**. **(a) **A schematic diagram of the CKAR activity reporter. **(b) **Live cell image of a mouse cardiomyocyte expressing the CKAR reporter [20]. AKAP79 targets the associated CKAR in proximity to the KCNQ2 subunit of the M current. CKAR permits rapid and transient FRET changes in response to changes in intracellular Ca^2+^.

Targeting of the PKC activity reporter CKAR to various intracellular membranes (plasma membrane, Golgi, mitochondrial outer membrane) has provided much insight into the rate, magnitude, and duration of agonist-evoked PKC signaling throughout the cell [9]. In a study that exemplifies the role of scaffolds in driving PKC function, CKAR was fused to the anchoring protein AKAP79 [20]. This anchored reporter was used to monitor the activation dynamics of PKC and concomitant changes in conductance through one of its substrates, the KCNQ2 subunit of the M current (Figure 4). Two important pieces of information emerged from these studies. First, PKC activation lagged 11 seconds behind changes in channel conductance when cytoplasmic CKAR was used as the reporter. However, fusion with AKAP79 to target the reporter to the ion channel, thereby anchoring PKC close to a substrate, abolished this lag: in this configuration, PKC activation and suppression of KCNQ2 currents occurred simultaneously. Second, proximity to AKAP79 abolished PKC sensitivity to ATP competitive inhibitors such as BIS-I. This unforeseen observation indicated that AKAP79 not only controls access to substrates but also influences how binding partners such as PKC respond to certain pharmacological inhibitors [20]. The finding that anchored kinases are refractory to active site inhibitors has important ramifications for drug discovery as endogenous binding partners that confer resistance to ATP analog inhibitors. Similar mechanisms could explain why cancers involving activated PKB and B-Raf can become resistant to therapeutics such as A-443654 and PLX4032, respectively, as they are active site inhibitors [21-25].

**Figure 5:**
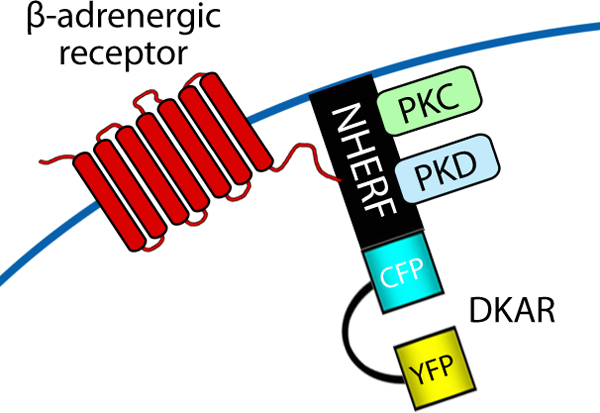
**Fusion of DKAR to a protein kinase D activation scaffold**. A schematic diagram of the DKAR activity reporter. Fusion of DKAR with NHERF permits monitoring of PKD activation at this protein scaffold.

Protein scaffolds also serve as a nexus for signaling by PKD. Of the three PKD isoforms, PKD1 and PKD2 have distinct PDZ ligands that bind the PDZ domains of NHERF, a family of scaffolds that coordinates signaling by ion channels, G-protein coupled receptors, and tyrosine kinase receptors [26]. Fusion of DKAR, onto the amino terminus of NHERF (Figure 5) has revealed a distinct signaling profile at the protein scaffold: the agonist-evoked activity of PKD is sustained on the protein scaffold, compared to cytosol or plasma membrane, where activation is transient [27]. Underscoring NHERF as a nexus for PKD signaling, the use of an isozyme-specific PKC activity reporter, δCKAR, which reports PKC δ activity [28], revealed amplified signaling of this kinase at the scaffold; this isozyme is the activating upstream kinase for PKD. These activity reporters have also been utilized to monitor the activation mechanism of PKD in cardiomyocytes, which involves its release from association with AKAP-lbc and translocation into nucleus [10].

## The expanding range of biosensors

A number of variants on FRET technology continue to expand our toolbox for visualizing signaling. One such variation is bioluminescent resonance energy transfer (BRET), which has been used to show dynamic interactions between the insulin receptor and the scaffold Grb14 [29]. Another is time-resolved luminescence resonance energy transfer (LRET), which has been used to monitor protein interactions in cells; this technique takes advantage of a luminescent terbium complex and luminescent energy transfer to GFP [30]. Each of these methods builds on the concept that a biological change alters a physical property that can be detected by appropriately engineered light-emitting probes.

In conclusion, the use of FRET-based reporters has allowed unprecedented insight into the cellular dialect of phosphorylation on protein scaffolds and membrane microdomains. By coordinating enzymes that propagate and terminate signaling, such macromolecular assemblies afford exquisite control of signaling in time and space. With the development of dual FRET pairs, the field is now poised to visualize the simultaneous activity of two kinases [31-33]. These dual FRET pairs have been optimized by systematic mutagenesis of chromo proteins to yield sufficient spectral separation to resolve FRET at two different wavelengths. Alternatively, one FRET acceptor can be used to monitor interactions by two different donors [34]. If reporters incorporating these FRET pairs are targeted to different subcellular compartments, it becomes theoretically possible to sample the activities of two kinases simultaneously. No doubt that exciting new discoveries about dialects of localized kinase activity and compartmentalized second messenger action will be revealed in years to come.
